# Optimal stimulation toward the dermal papilla lineage can be promoted by combined use of osteogenic and adipogenic inducers

**DOI:** 10.1002/2211-5463.12763

**Published:** 2019-12-28

**Authors:** Taheruzzaman Kazi, Ichitaro Niibe, Akio Nishikawa, Takashi Matsuzaki

**Affiliations:** ^1^ Bioscience and Biotechnology The United Graduate School of Agricultural Sciences Tottori University Japan; ^2^ Department of Biological Science Faculty of Life and Environment Science Shimane University Japan

**Keywords:** adipogenic, dermal papilla cells, hair regeneration, osteogenic, versican

## Abstract

Dermal papilla cells (DPCs) play crucial roles in hair regeneration, but they readily lose their hair‐forming ability during *in vitro* culture. Although the formation of spheroids partially restores the ability, shrinkage of the spheroids makes it difficult to maintain cellular viability. To address this problem, we stimulated DPCs with factors known to induce adipogenic and/or osteogenic differentiation, because DPCs share unique gene expression profiles with adipocytes and osteocytes. We isolated DPCs from versican (*vcan*)–GFP mice, in which GFP is expressed under the control of a *vcan* promoter, which is strongly active in DPCs of anagen hair follicles. GFP fluorescence was most intense when the spheroids were made from DPCs cultured in a half‐diluted combination of adipogenic and osteogenic media (CAO1/2), a Dulbecco’s modified Eagle’s medium‐based medium that contains 10% FBS, 275 nm dexamethasone, 2.5 mm β‐glycerol phosphate, 12.5 µg·mL^−1^ ascorbic acid, 0.125 µm isobutylmethylxanthine and 2.5 ng·mL^−1^ insulin. The dose of each additive used was less than the optimal dose for adipogenic or osteogenic differentiation, and shrinkage of the spheroids was avoided through the addition of fibroblast growth factor 2 and platelet‐derived growth factor‐AA to  CAO1/2. In addition, the gene and protein expression of *vcan*, osteopontin, alkaline phosphatase and α‐smooth muscle actin in the spheroids were augmented to levels similar to those of the intact dermal papillae, which exhibited restored hair‐forming activity. In conclusion, a combination of certain adipogenic and osteogenic inducers, together with fibroblast growth factor 2 and platelet‐derived growth factor‐AA, can promote differentiation toward the DPC lineage.

## Graphical abstract

Dermal papilla cells (DPCs) were isolated from versican–GFP mice. Green fluorescence (indicative of hair‐forming activity) and DPC‐specific gene expression were enhanced in spheroids derived from DPCs when they were cultured with both adipogenic and osteogenic differentiation factors at moderate concentrations in addition to fibroblast growth factor 2 and platelet‐derived growth factor‐AA (half‐diluted combination of adipogenic and osteogenic media). Hair neogenesis was observed only in nude mice grafted with these spheroids.

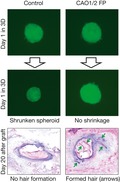

**Abbreviations**
2D: two‐dimensional
3D: three‐dimensional
ALPL: alkaline phosphatase
ASMA: α‐smooth muscle actin
CAO: combination of adipogenic and osteogenic media
CAO1/2: half‐diluted CAO
CAO1/2‐FP: CAO1/2 with FGF2 and PDGF‐AA
DMEM: Dulbecco’s modified Eagle’s medium
DP: dermal papilla
DPC: dermal papilla cell
ECM: extracellular matrix
FGF2: fibroblast growth factor 2
HSD: honestly significant difference
IBMX: isobutylmethylxanthine
MSC: mesenchymal stem cell
Opn: osteopontin
PDGF‐AA: platelet‐derived growth factor‐AA
PPARγ: peroxisome proliferator‐activated receptor γ
qPCR: quantitative PCR
vcan: versican

Mammalian hair follicles contain specialized mesenchymal cells at the proximal end of the hair bulb region known as the dermal papilla cells (DPCs), which play pivotal roles in the differentiation and proliferation of the hair matrix cells 1. The founder cells of DPCs differentiate and aggregate through the sequential crosstalks with the overlaying epithelium in the developmental process of the hair follicles 2. These aggregates are indispensable not only for embryonic hair follicle development, but also in postnatal hair cycling 3, 4. In fact, the intact dermal papillae (DPs) can induce hair follicles *de novo* in the recipient skin, even if the epidermis has been derived from a non‐hair‐bearing region 5. Therefore, DPCs have been thought to possess a strong hair‐forming ability; however, their nature is not clear yet.

DPs contain a vast amount of the extracellular matrix (ECM) proteins such as versican (*vcan*) and perlecan, as well as laminin and collagen types I, III and IV 6, 7. *vcan* is the core protein of a multifunctional chondroitin sulfate proteoglycan 8. It inhibits many types of cell–substratum adhesion, by which it may control cell proliferation and differentiation in organogenesis 9. The amount of *vcan* in a DP increases in the anagen phase (the active growth phase of hair follicles) and reaches the maximum level; then, the expression rapidly decreases in the catagen phase (the regression phase) and is abolished by the end of the telogen phase (the resting phase) 10, 11. An anagen DP is the largest compared with those in other stages, because *vcan* and other ECM proteins are actively secreted in anagen and deposited in the intercellular space between the DPCs.

Once the DPs are isolated from the body and outgrown *in vitro*, DPCs rapidly lose their hair‐forming ability upon culturing beyond five rounds of subcultivation 12. Similarly, DPCs with low passage numbers express *vcan* at an intense level, but those with high passages tend to lose its expression 13. DPCs can maintain *vcan* expression upon continuous stimulation with appropriate growth factors, such as fibroblast growth factor 2 (FGF2) and platelet‐derived growth factor (PDGF) 14. Moreover, the hair follicle inductivity can be partially restored in a three‐dimensional (3D) culture 15. Kishimoto *et al*. 13 generated *vcan*–GFP transgenic mice that express the GFP under the control of the *vcan* promoter. The GFP expression was prominent in DPCs of these mice in the anagen phase. They had clearly demonstrated the close relationship between the GFP fluorescence intensity and the hair inductivity of the DPCs that had been derived from the *vcan*–GFP mice 13. Therefore, the hair‐forming activity of the DPCs can be visually monitored using the *vcan*–GFP mice.

DPCs are thought to derive from a mesenchymal lineage in the early stage of hair follicle development in embryos 3. Mesenchymal stem cells (MSCs) and their descendants are known to differentiate into various cell types depending on stimulation from the cellular environment. For example, MSCs differentiate into osteocytes when they are stimulated with 100 nm dexamethasone, 10 mm β‐glycerol phosphate and 50 µg·mL^−1^ ascorbic acid 16. Upon treatment of 3T3L1 fibroblasts with 1 µm dexamethasone, 0.5 mm isobutylmethylxanthine (IBMX) and 10 μg·mL^−1^ insulin, they differentiate into adipocytes 17. Although the founder cells of DPCs are still unclear, DPCs share unique gene expression profiles with that of adipocytes and osteocytes, such as leptin and osteopontin (*opn*), respectively. In addition, the DPCs drastically change their hair‐forming ability during the different hair cycle stages. Alkaline phosphatase (*alpl*) is also a typical DP‐specific maker gene. *Alpl* is expressed intensely in the dermal sheath cells covering the bottom area of the hair bulb; its expression begins in the very early stage of the anagen phase, reaches highest level in the mid‐anagen phase, and rapidly ceases in the catagen phase 18. Because cultured DPCs without the *alpl* expression lose their hair‐forming ability, *alpl* appears to be closely related to DP‐specific functions. These facts suggest that the DPCs are refreshed and recover their hair inductivity in the early anagen phase in each hair cycle, by responding to differentiation factors from the surrounding cells, some of which might be similar to the inducers of the adipogenic or osteogenic differentiation from MSCs. Here, we examined whether the combination of adipogenic and osteogenic factors promotes DP‐specific characteristics in cultured DPCs using the *vcan*–GFP transgenic mice and 3D culture techniques.

## Materials and methods

### Media

Dulbecco’s modified Eagle’s medium (DMEM)‐based adipogenic and osteogenic media were mixed at a 1 : 1 ratio and referred to as combination of adipogenic and osteogenic media (CAO), which contains 10% FBS, 550 nm dexamethasone (the usual working dose is 1000 nm for adipogenic and 100 nm for osteogenic; MP Biomedicals, Irvine, CA, USA), 0.5 µm IBMX (adipogenic; Fujifilm Wako Pure Chemical, Osaka, Japan), 10 μg·mL^−1^ insulin (adipogenic; Sigma‐Aldrich, St Louis, MO, USA), 10 mm β‐glycerol phosphate (osteogenic; TCI, Tokyo, Japan) and 50 µg·mL^−1^ ascorbic acid (osteogenic; Fujifilm Wako). CAO was further diluted to twice the volume with DMEM containing 10% FBS and referred to as half‐diluted CAO (CAO1/2). In certain experiments, DMEM or CAO1/2 was supplemented with FGF2 and/or PDGF‐AA (PeproTech, Rocky Hill, NJ, USA) at different concentrations.

### Isolation and culture of DPCs

*vcan*–GFP mice, the *vcan* promoter‐driven GFP‐expressing mice, were kindly provided by J. Kishimoto (Shiseido, Japan). DPs were isolated from the vibrissa follicles, in anagen phase, of the *vcan*–GFP mice and cultured in papilla cell growth medium (Toyobo, Osaka, Japan) in an atmosphere of 5% CO_2_ at 37 °C. Outgrowing DPCs were collected by using Accutase (Innovative Cell Technologies, San Diego, CA, USA) and subcultured up to passage 5 (P = 5) to propagate the cell number; then, they were aliquoted and preserved in liquid N_2_. Thawed DPCs were aliquoted and cultured in CAO, CAO1/2 or DMEM in a six‐well culture plate at 6 × 10^4^ cells per well. After 5 days, the cells were dissociated with Accutase and inoculated in a low‐adhesive 96‐well plate (Prime surface; Sumitomo Bakelite, Tokyo, Japan) at 3 × 10^3^ cells per well to prepare a 3D structure known as the spheroid, for 4 or 5 days. All procedures performed in the studies involving animals were in accordance with the ethical standards of the Animal Research Committee of Shimane University, where the studies were conducted.

### Measurement of spheroid characteristics

Fluorescent micrographs of each spheroid were taken every day using an inverted fluorescence microscope (Nikon Eclipse T*i*; Nikon, Tokyo, Japan). *vcan* expression in the spheroids was indicated by the GFP fluorescence intensity, and the spheroid size was estimated from the projected area of the micrographs using the imagej public domain software (NIH, Bethesda, MD, USA). Statistical analyses were performed using the *t*‐test on six spheroids from each experimental group.

### RNA extraction and quantitative PCR

Total RNA was obtained from the spheroids on day 4 and from the intact DPs of vibrissa follicles, as the positive control, using the RNeasy mini kit (Qiagen, Germany); cDNAs were prepared from the total RNA using the QuantiTect reverse transcription kit (Qiagen, Hilden, Germany). Quantitative PCR (qPCR) analysis was performed using the Thermal Cycler Dice® Real Time System (TP800; Takara Bio, Kusatsu, Japan) with the cDNAs and Syber Premix Ex Taq II kit (Takara Bio), to examine the gene expression levels of *vcan*, *opn*, α‐smooth muscle actin and *alpl*. The expression level of the *GAPDH* gene was used as the internal control in all of the experiments.

### Whole‐mount immunocytochemistry

Lab‐Tek II chamber slides (eight wells per slide; Thermo Fisher Scientific, Waltham, MA, USA) were coated with 30 μL·well^−1^ of 0.5 mg·mL^−1^ collagen I (IPC‐50; Koken, Tokyo, Japan) in PBS until drying up. The spheroids of C57BL/6‐derived DPCs were made as above with CAO1/2 or DMEM and transferred into the chamber slides using wide‐bore pipette tips. After the spheroids attached to the surface, the culture medium was carefully removed. The spheroid was covered again with 30 μL·well^−1^ of 0.5 mg·mL^−1^ collagen I to prevent peeling off from the surface during immunocytochemistry. Then, the spheroids were fixed with 4% paraformaldehyde for 10 min and permeabilized with 1% Triton X‐100 for 10 min at room temperature. Then, they were blocked for 30 min with Block Ace (DS Pharma Biomedical, Osaka, Japan) and treated with anti‐ASMA Ig (MAB1420; R&D Systems, Minneapolis, MN, USA) or anti‐ALPL Ig (AF2910; R&D Systems) at manufacturer’s recommended dilution in PBS at 4 °C for overnight. They were thoroughly washed with PBS containing 0.05% Tween 20 and reacted with Alexa Flour 488‐ or Alexa Flour 594‐conjugated secondary antibodies at 1 : 800 dilution. DAPI Fluoromount G (SouthernBiotech, Birmingham, AL, USA) was used for nuclei stain. Fluorescent micrographs were taken under a fluorescence microscope (BX51N; Olympus, Tokyo, Japan) aided with a digital monochrome camera (DFC345FX; Leica Microsystems, Wetzlar, Germany). The fluorescent intensities were measured for three spheroids for each category using imagej software and averaged.

### Biological assay

Patch assay was carried out according to Zheng *et al*. 19 with the C57BL/6‐derived DPCs of passage 5. The cells were cultured in CAO1/2 supplemented with both FGF2 and PDGF‐AA or DMEM for 7 days in monolayer culture. Then, 0.75 × 10^6^ DPCs and 1.5 × 10^6^ newborn‐derived keratinocytes were mixed and inoculated into a hanging drop culture plate (Elplasia MPs500; Kuraray, Tokyo, Japan), which contains 680 micropores for making spheroids. Thus, one spheroid included ~ 1100 DPCs and 2200 keratinocytes. The spheroids were centrifuged and concentrated in 10–12 μL cell suspension and were inserted into the hypodermis of host nude mice. The patches formed underneath the skin were collected on 31 or 34 days after the transfer, embedded in OCT compound and frozen. Thin sections, 5 μm thick, were made with Leica CM 1510S cryostat and fixed with 10% neutral formalin for 10 min. The samples were stained in Mayer’s hematoxylin and eosin solutions and observed under a Keyence BZ‐X700 microscope (Keyence, Osaka, Japan).

## Results

### CAO promotes the expression of DPC‐related genes

**Figure 1:**
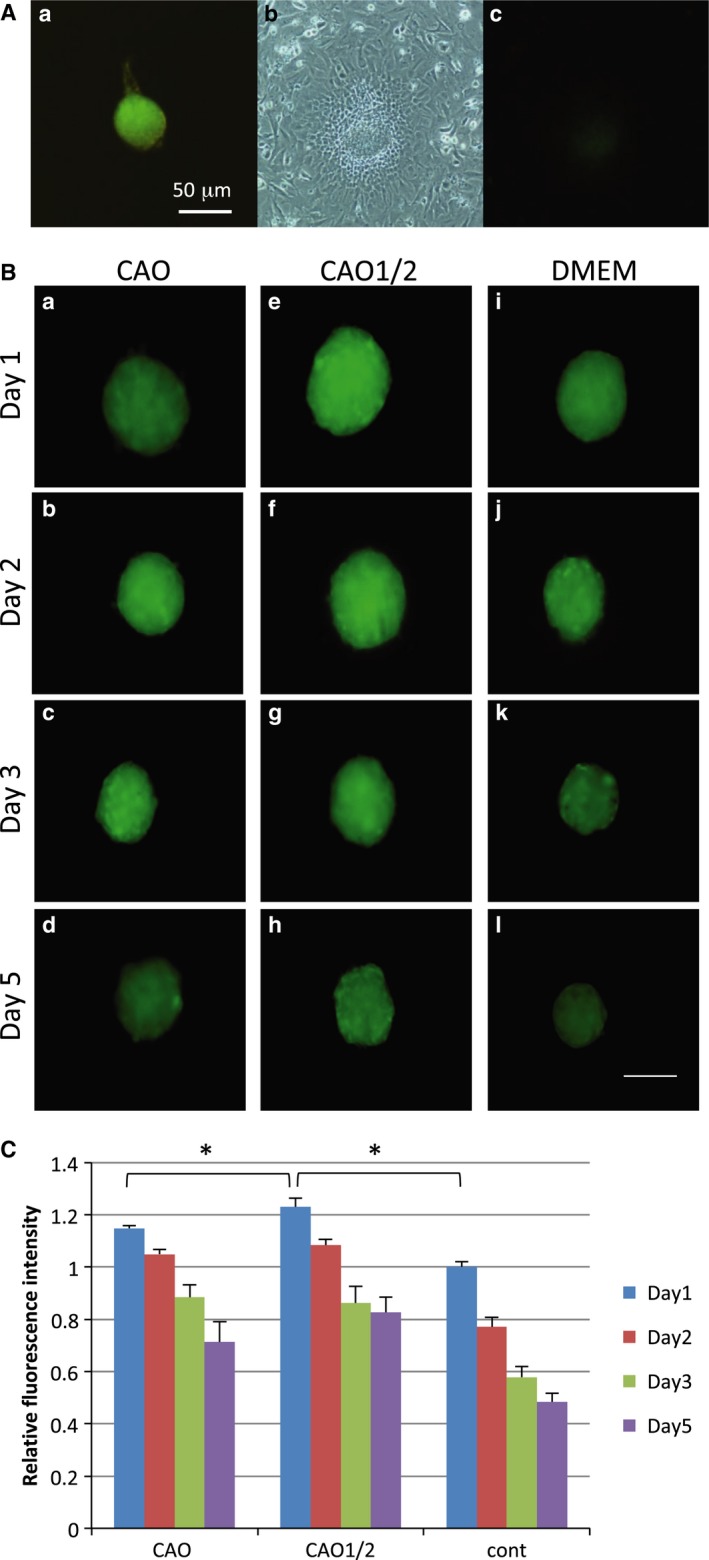
*vcan* expression was stimulated in DPCs cultured with the adipogenic and osteogenic inducers. DPs were isolated from the vibrissa follicles of the *vcan*–GFP transgenic mice in the anagen phase, which showed intense GFP fluorescence as an indication of the expression level of the *vcan* gene (Aa). The DPCs outgrown *in vitro* were reduced in their fluorescence (Ab, Ac). The DPCs were passaged five times in the control medium (DMEM) and were subcultured in the CAO (Ba‐Bd), CAO1/2 (Be–h) or control (Bi–l) media for 5 days. Then, the DPCs were inoculated at 3 × 10^3^ cells per well in a low‐adhesive 96‐well plate to make a spheroid and cultured for 5 more days. The fluorescence micrographs of six spheroids from each category were taken at days 1 (Ba, e, i), 2 (Bb, f, j), 3 (Bc, g, k) and 5 (Bd, h, l), from which the GFP fluorescence intensities were calculated by image analyses (C). GFP fluorescence at day 5 was strongest in the spheroids cultured in CAO1/2 and rapidly decreased in those cultured in the control medium (cont). Scale bars, 50 μm (A); 100 μm (B). Vertical bars indicate standard deviation (C). The difference was assessed using ANOVA with *post hoc* Tukey’s honestly significant difference (HSD) test. Significant differences to CAO1/2 on day 1 are indicated as *P > 0.05.

**Figure 2:**
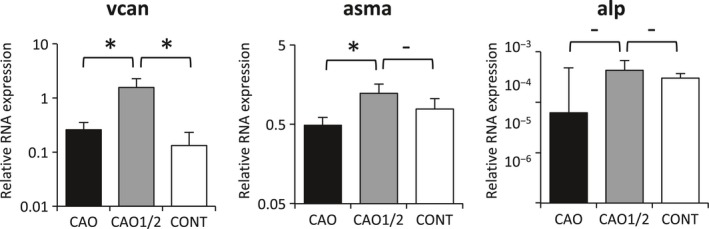
Expression of DPC‐related genes in the spheroids. Total RNA was isolated from the spheroids cultured in CAO, CAO1/2 or DMEM at day 4 of the 3D culture, and cDNAs were prepared from them. The expression levels of DPC‐related genes were estimated by qPCR using the cDNA and the specific primer sets for *vcan*, ASMA (*asma*) and *alpl*. Vertical bars indicate standard deviation. The difference was assessed using ANOVA with *post hoc* Tukey’s HSD test. Significant differences to CAO1/2 are indicated as *P < 0.05.

We used the *vcan*–GFP mice to visually indicate the hair‐inductive property of DPCs, because the expression level of *vcan* is closely related to the extent of the property. The GFP fluorescence was the strongest in anagen DPs (Fig. 1Aa), which rapidly diminished when they were cultured *in vitro* (Fig. 1Ab,c). It is well known that the gene expression profiles are vastly different between the cells maintained in the two‐dimensional (2D) monolayer culture and the 3D spheroid culture, and that the profile in the latter is similar to those of intact tissues. The *vcan* expression was recovered by preparing spheroids of the DPCs in the low‐adhesive culture plate, but it decreased in a few days when cultured in DMEM (Fig. 1Bi–l). To enhance the activity of DPCs, we cultured the cells under a combination of adipogenic and osteogenic factors at different concentrations (CAO or CAO1/2) for 5 days and prepared spheroids from these cells. By adding these factors, the GFP fluorescence was augmented in the spheroids of DPCs that had been cultured in CAO (CAO spheroids; Fig. 1Ba–d) or CAO1/2 (CAO1/2 spheroids; Fig. 1Be–h), compared with the control spheroids (Fig. 1Bi–l). Then, the GFP fluorescence intensity was measured for each spheroid by image analyses using the imagej software. The fluorescence was more intense in the CAO or CAO1/2 spheroids in comparison with the control on day 1, which was the day after initiating the spheroid preparation in the low‐adhesive culture plate (Fig. 1C). On day 5 of the 3D culture, the GFP fluorescence in the control spheroids was reduced to 52%, but the CAO1/2 spheroids retained the fluorescence at more than 67% of the initial value (Fig. 1C). The spheroids gradually shrank during the 3D culture because the cells contacted and pulled each other, which was clearly seen as the decrease in the spheroid volume (Fig. 1B). The size reduction was more prominent in the control spheroids than in the CAO or CAO1/2 spheroids. In the intact DPs, the intercellular space is filled with a vast amount of ECM proteins, such as *vcan*. The ECM may avoid the shrinkage of the DP *in vivo*, as well as transduce the microenvironmental signals to the DPCs. qPCR revealed that the expression of *vcan* was the highest in the CAO1/2 spheroids (Fig. 2). In addition, the other two DP‐related genes, ASMA (*asma*) and tissue‐nonspecific *alpl*, were expressed at a higher level in the CAO1/2 spheroids (Fig. 2). Interestingly, the CAO spheroids expressed the DPC‐related genes at similar levels to the control (Fig. 2), although their GFP fluorescence and size were comparable with the CAO1/2 spheroids (Fig. 1B,C). These results suggest that the CAO1/2 medium would be effective in maintaining the DPC‐specific properties in the spheroids, but not enough to avoid shrinkage of the spheroids during the 3D culture.

### FGF2 and PDGF‐AA prevent spheroid shrinkage and retain *vcan* expression

**Figure 3:**
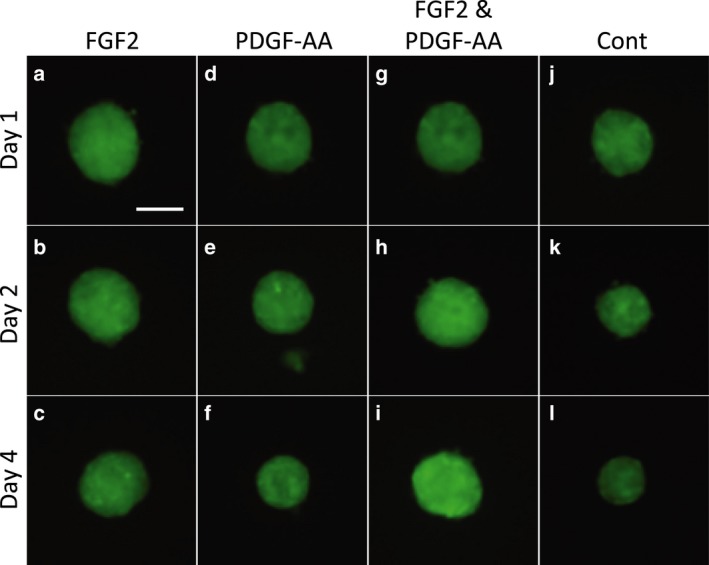
FGF2 and PDGF‐AA prevented the shrinkage of DPC spheroids. Spheroids of the *vcan*–GFP mouse‐derived DPCs shrank during the 3D culture for 4 days in the control medium DMEM (j–l). The shrinkage was prevented by the addition of FGF2 in CAO1/2 (a–c, g–i), but not by addition of PDGF‐AA alone (d–f). Interestingly, GFP fluorescence was augmented in the spheroids cultured in CAO1/2 supplemented with both FGF2 and PDGF‐AA compared with in those stimulated with one of these factors alone. Scale bar, 100 μm.

**Figure 4:**
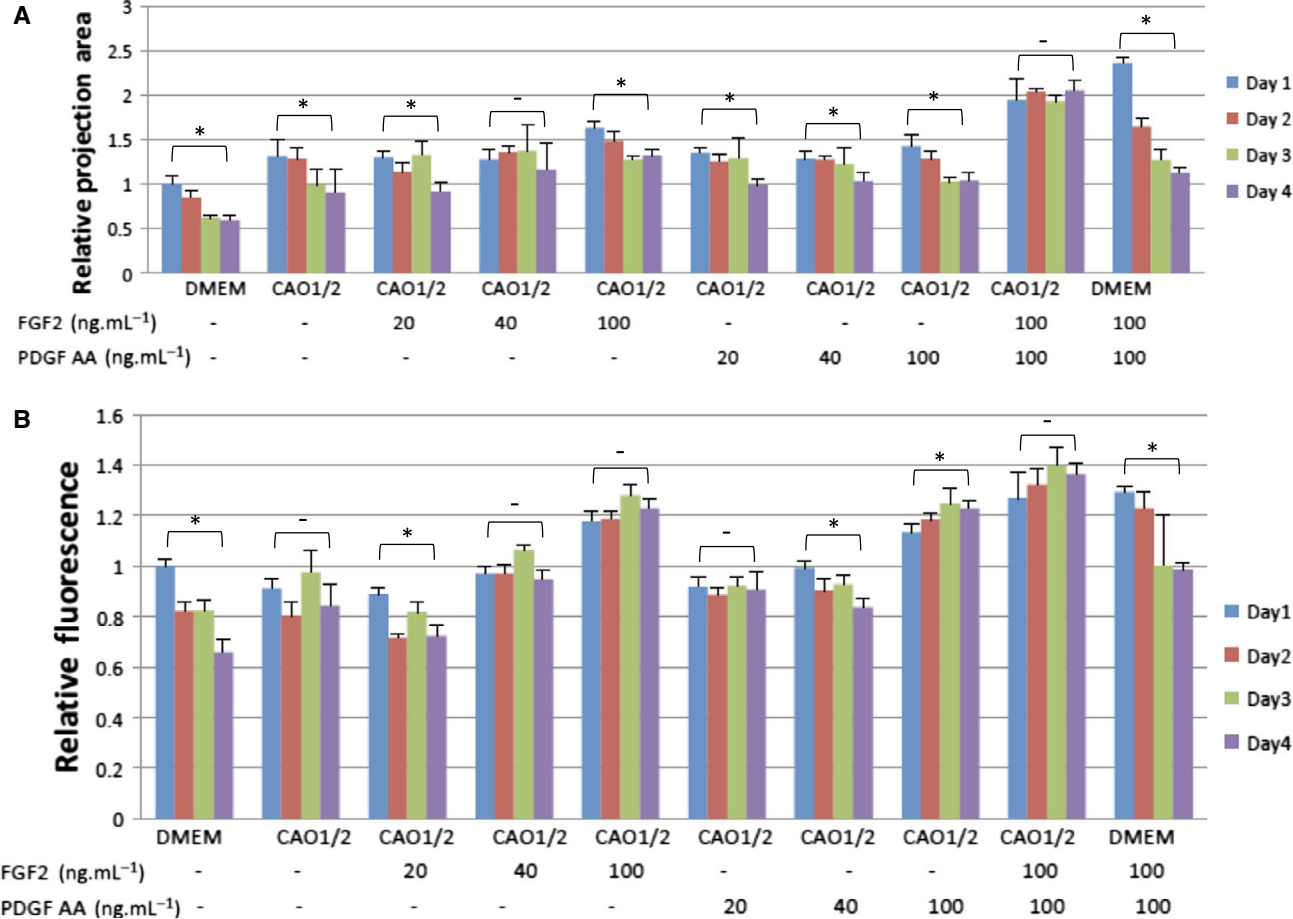
*vcan* expression and the volume of the DPC spheroids were promoted by the addition of FGF2 and PDGF‐AA during the 3D culturing. DPCs spheroids were cultured in the CAO1/2 or control DMEM, with or without FGF2 and PDGF‐AA at 20, 40 or 100 μg·mL^−1^. The size of the spheroids was analyzed from fluorescence micrographs (A). The size is indicated as its projection area. The average size of three spheroids was the largest in those stimulated with both FGF2 and PDGF‐AA in CAO1/2, which could be retained at its initial value for at least 4 days. In contrast, the spheroids cultured in DMEM shrank drastically. The *vcan* expression was estimated from the GFP intensity by image analyses (B). The *vcan* expression was promoted in the DPC spheroids by stimulation with FGF2 or PDGF‐AA in a dose‐dependent manner. The factors showed a synergistic effect on the upregulation of *vcan* gene expression and could maintain the expression level for at least 4 days. The *vcan* expression was also stimulated in the spheroids cultured in DMEM with FGF2 and PDGF‐AA, but it dropped within 3 days in the 3D culture. Vertical bars indicate standard deviation. Statistical analyses were performed using the *t*‐test between days 1 and 4 in each experimental group. The difference was assessed using ANOVA with *post hoc* Tukey’s HSD test. Significant differences are indicated as *P < 0.05.

It has been previously reported that FGF2 and PDGF‐AA are positive factors for DPC proliferation *in vitro*. Thus, we examined the effect of both factors on DPC spheroids, in combination with CAO or CAO1/2. FGF2 and PDGF‐AA were added at three different concentrations, namely, 20, 40 and 100 ng·mL^−1^. DPCs were cultured for 5 days and inoculated into a low‐adhesive culture plate at 3000 cells per well, containing the same medium as 2D culture, to prepare the spheroids. Under the 3D condition, the control spheroids, which had been produced from DPCs cultured in DMEM, drastically shrank in 2 days (Figs 3j3, 4l and 3, 4A). Interestingly, the spheroids retained their volume and *vcan* expression when FGF2 and PDGF‐AA had been added to the CAO1/2 medium (Fig. 3g–i), although these factors did not show such positive effects with CAO (data not shown). Image analyses revealed that FGF2 or PDGF‐AA alone could enhance the *vcan* expression at 100 ng·mL^−1^, but not efficiently at 40 ng·mL^−1^ or less, as far as judging from the GFP fluorescence (Fig. 4B); however, none of them individually could avoid the size reduction of the spheroids (Fig. 4A). In contrast, the spheroids cultured in both FGF2 and PDGF‐AA, at 100 ng·mL^−1^ each, did not show any shrinkage (Fig. 4A). The GFP fluorescence in these spheroids was stronger than the others, which showed a tendency to increase during cultivation for 4 days without medium change (Fig. 4B). Adding FGF2 and PDGF‐AA at 100 ng·mL^−1^ each into the control DMEM also increases the volume of the spheroids and the GFP fluorescence, but such stimulating effects rapidly diminished within 3 days (Fig. 4). These results suggest that the combination of FGF2 and PDGF‐AA at higher concentrations would promote a vast amount of ECM molecules such as *vcan*, which supports the construction and maintenance of the cohesive and flexible 3D structure of the DP.

### Synergistic effects of FGF2 and PDGF‐AA with CAO1/2 medium on the expression of DPC‐related genes

**Figure 5:**
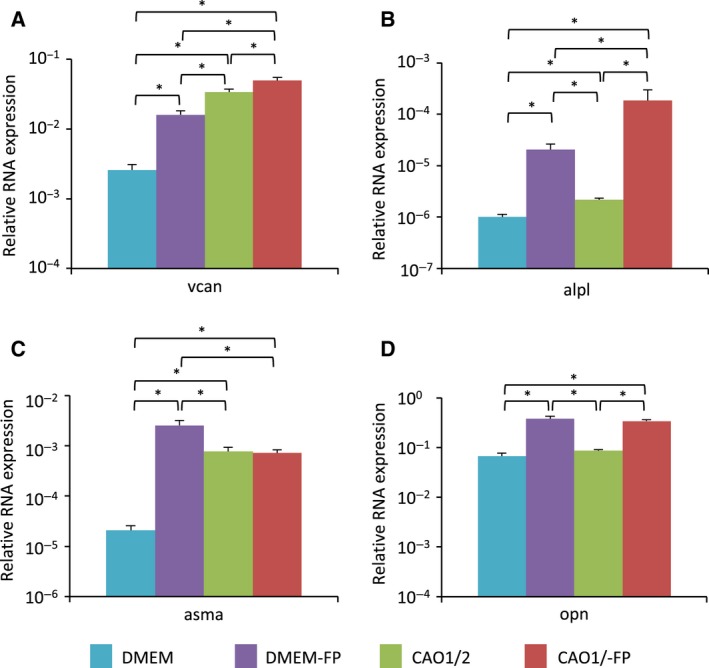
Expression levels of the DPC‐related genes in the DPC spheroids. Spheroids were prepared in CAO1/2 medium or in the control DMEM, with or without FGF2 and PDGF‐AA. The total RNAs were extracted from the spheroids on day 4 of the 3D culture and converted to cDNAs, which were used for qPCR analyses with specific primer sets for the DPC‐related genes, *vcan* (A), *alpl* (B), *asma* (C) and *opn* (D). The average gene expression for each category was obtained from two independent experiments with triplicate measurements and shown with a vertical bar indicating standard deviation. The difference was assessed using ANOVA with *post hoc* Tukey’s HSD test. Significant differences are indicated as *P < 0.05.

Next, we examined the effects of FGF2 and PDGF‐AA on the expression of DPC‐related genes in the DPC spheroids. The DPC spheroids cultured in DMEM expressed the *vcan* gene vigorously when they were stimulated with both FGF2 and PDGF‐AA at 100 ng·mL^−1^ (DMEM‐FP spheroids; Fig. 5A). Although the average *vcan* expression was very high in the spheroids cultured in CAO1/2 medium even without FGF2 and PDGF‐AA stimuli, it could be further promoted by these factors [CAO1/2 with FGF2 and PDGF‐AA (CAO1/2‐FP) spheroids; Fig. 5A]. FGF2 and PDGF‐AA intensely augmented the expression of the *alpl* gene in the spheroids regardless of the type of media (Fig. 5B). The *alpl* expression in the CAO1/2‐FP spheroids was ~ 10‐fold more than that in the DMEM‐FP spheroids and 200‐fold more than that in the DMEM spheroids. FGF2 and PDGF‐AA intensely induced the *asma* gene expression in the spheroids cultured in DMEM, but they did not show additional effects in those cultured in the CAO1/2 medium, which could vigorously increase the gene expression in comparison with the DMEM (Fig. 5C). The expression of an osteocyte marker gene, *opn*, was also stimulated more than 5‐fold in the DPC spheroids that were treated with FGF2 and PDGF‐AA compared with those without the stimuli, but no difference was observed between the DMEM‐FP spheroids and CAO1/2‐FP spheroids (Fig. 5D). These results suggest that the synergistic effect of these factors and CAO1/2 medium is obvious on *vcan* and *alpl* expressions.

**Figure 6:**
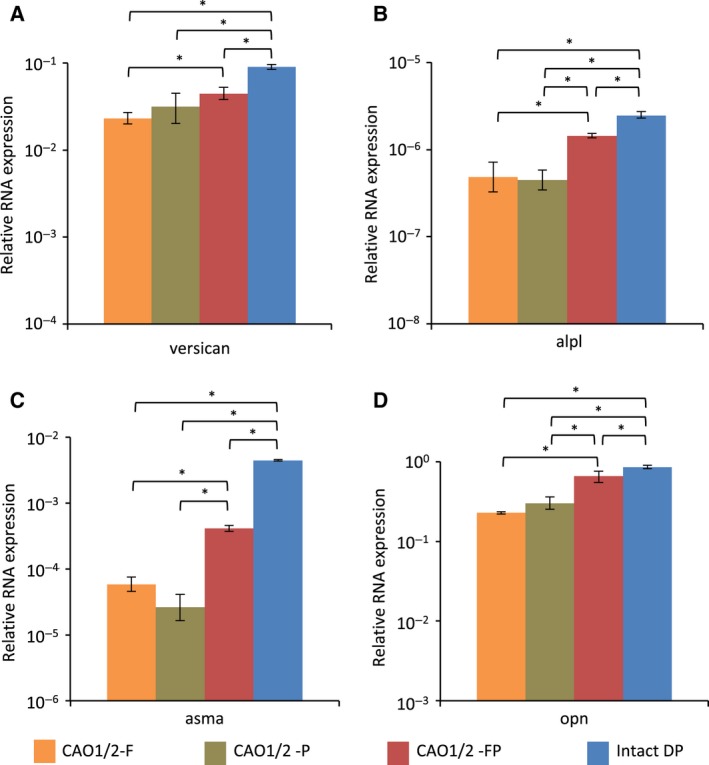
Comparison of the expression of the DPC‐related genes in DPC spheroids with that in intact papillae. Total RNA was extracted from the spheroids cultured under various conditions and from the intact DPs isolated from the vibrissa follicles. The total RNA was reverse transcribed and used in qPCR analyses of the DP‐related genes, *vcan* (A), *alpl* (B), *asma* (C) and *opn* (D). The average gene expression for each category was obtained from two independent experiments with triplicate measurements and shown with a vertical bar indicating standard deviation. The difference was assessed using ANOVA with *post hoc* Tukey’s HSD test. Significant differences are indicated as *P < 0.05.

As suggested by the GFP fluorescence shown in Fig. 3, the average *vcan* expression in the CAO1/2‐FP spheroids was higher than that in the spheroids treated with one of the factors, although a significant difference was not detected between the spheroids cultured in CAO1/2 with PDGF‐AA and the CAO1/2‐FP spheroids (Fig. 6A). The expression of the *alpl* gene was also stimulated in the CAO1/2‐FP spheroids to a level similar to that in the intact DPs (Fig. 6B). Similarly, FGF2 and PDGF‐AA showed synergistic effects on the *asma* expression in the spheroids cultured in the CAO1/2 medium (Fig. 6C). The expression of the *opn* gene was also enhanced in the CAO1/2‐FP spheroids to a comparable level with that in the intact DPs (Fig. 6D). Thus, both FGF2 and PDGF‐AA would be indispensable to promote DP‐specific characteristics under the 3D culture condition.

### CAO1/2 FP promoted ASMA and ALPL protein expression and stimulated the hair‐forming activity of DPC spheroids

**Figure 7:**
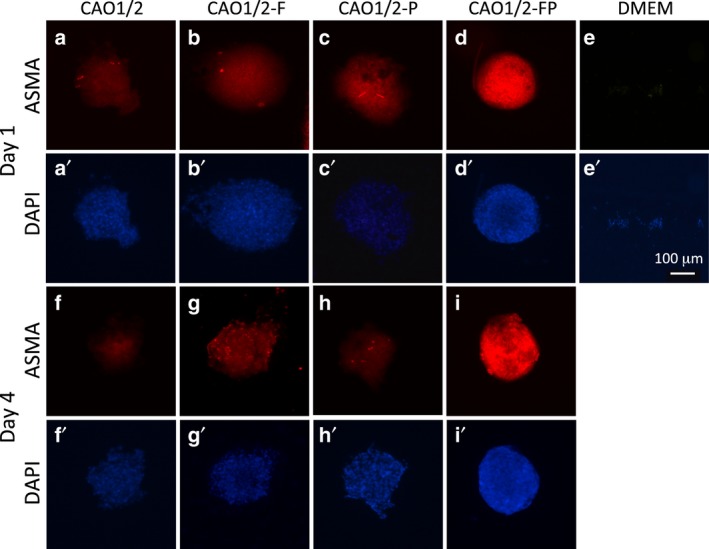
The effects of FGF2 and PDGF‐AA on ASMA expression in the DPC spheroids. The expression of ASMA protein was detected in the spheroids of the DPCs on days 1 (a–e) and 4 (f–i) of 3D culture by immunocytochemistry using anti‐ASMA Ig and Alexa Fluor 594‐conjugated secondary antibody. Nuclei were counterstained with DAPI (a′–i′). The fluorescence was weaker in the spheroids cultured with CAO1/2 only (a, f), CAO1/2‐F (b, g) or CAO1/2‐P (c, h) compared with that with CAO1/2‐FP (d, i). The spheroids of the DPCs cultured with DMEM were used as negative control, which showed a tendency to disperse during the antibody treatment (e). Scale bar, 100 μm.

**Figure 8:**
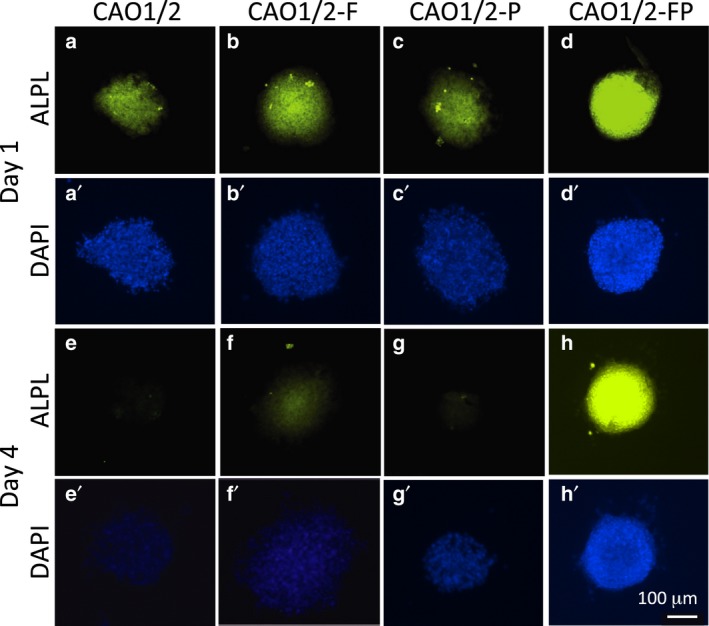
The effects of FGF2 and PDGF‐AA on ALPL expression in the DPC spheroids. The expression of ALPL protein was detected in the spheroids of the DPCs on days 1 (a–d) and 4 (e–h) of 3D culture by immunocytochemistry using anti‐ALPL Ig and Alexa Fluor 488‐conjugated secondary antibody. Nuclei were counterstained with DAPI (a′–h′). Similar to ASMA, the DPC spheroid cultured with CAO1/2‐FP (d) showed higher ALPL expression than those with CAO1/2 (a), CAO1/2‐F (b) or CAO1/2‐P (c). The expression of ALPL lasted for 4 days in the spheroids with CAO1/2‐FP (h) but quickly decreased in those with CAO1/2 (e), CAO1/2‐FGF2 (f) and CAO1/2‐PDGF‐AA (g). Scale bar, 100 μm.

**Figure 9:**
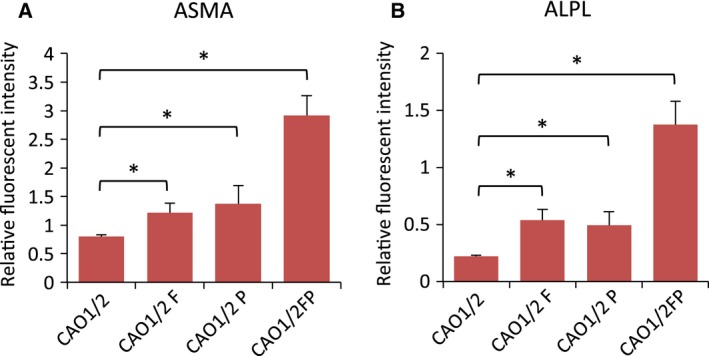
FGF2 and PDGF‐AA synergistically augment the expression of ASMA and ALPL in DPC spheroids. DPCs were cultured with CAO1/2 with or without FGF2 (F) or PDGF‐AA (P) and 3D cultured for 4 days. The protein expressions of ASMA and ALPL were visualized by whole‐mount immunocytochemistry with fluorescent dye‐conjugated antibodies as shown in Figs 7 and 8. The expression levels of ASMA (A) and ALPL (B) were estimated from the fluorescent intensities using ImageJ software. Vertical bars indicate standard deviation. The difference was assessed using ANOVA with *post hoc* Tukey’s HSD test. Significant differences to CAO1/2 are indicated as *P > 0.05.

We compared the expression levels of ASMA and ALPL protein among CAO1/2, CAO1/2‐F, CAO1/2‐P and CAO1/2‐FP spheroids by whole‐mount immunocytochemistry with fluorescent dye‐conjugated antibodies on days 1 and 4 of the 3D culture (Figs 7 and 8). Immunofluorescence of ASMA was the strongest in the CAO1/2‐FP spheroids on day 1 (Fig. 7d), and the expression lasted for at least 4 days (Fig. 7i). The other spheroids showed only a weak fluorescence. The control spheroids made of DPCs cultured in DMEM tended to collapse, and most cells were dispersed during immunocytochemistry (Fig. 7e). The expression of ASMA protein in the CAO1/2‐FP spheroids was 4‐fold higher than that in the CAO1/2 spheroids on average on day 4 (Fig. 9A). The ALPL expression in the spheroids was similar to those of ASMA (Fig. 8). The CAO1/2‐FP spheroids showed intense immunofluorescence for ALPL (Fig. 8d,h), which was 6‐fold stronger than that of the CAO1/2 spheroids on day 4 (Fig. 9B). In addition to the strong expression of ASMA and ALPL, the CAO1/2‐FP spheroids sustained their globular and cohesive structure, whereas other spheroids tended to be loosened or dissociated in 4 days. Although FGF2 or PDGF‐AA alone could promote the expression of ASMA and ALPL protein (Fig. 9), the synergistic effects of the factors were clearly shown on expression of DPC‐related proteins and also sustaining the spheroid structure and function.

**Figure 10:**
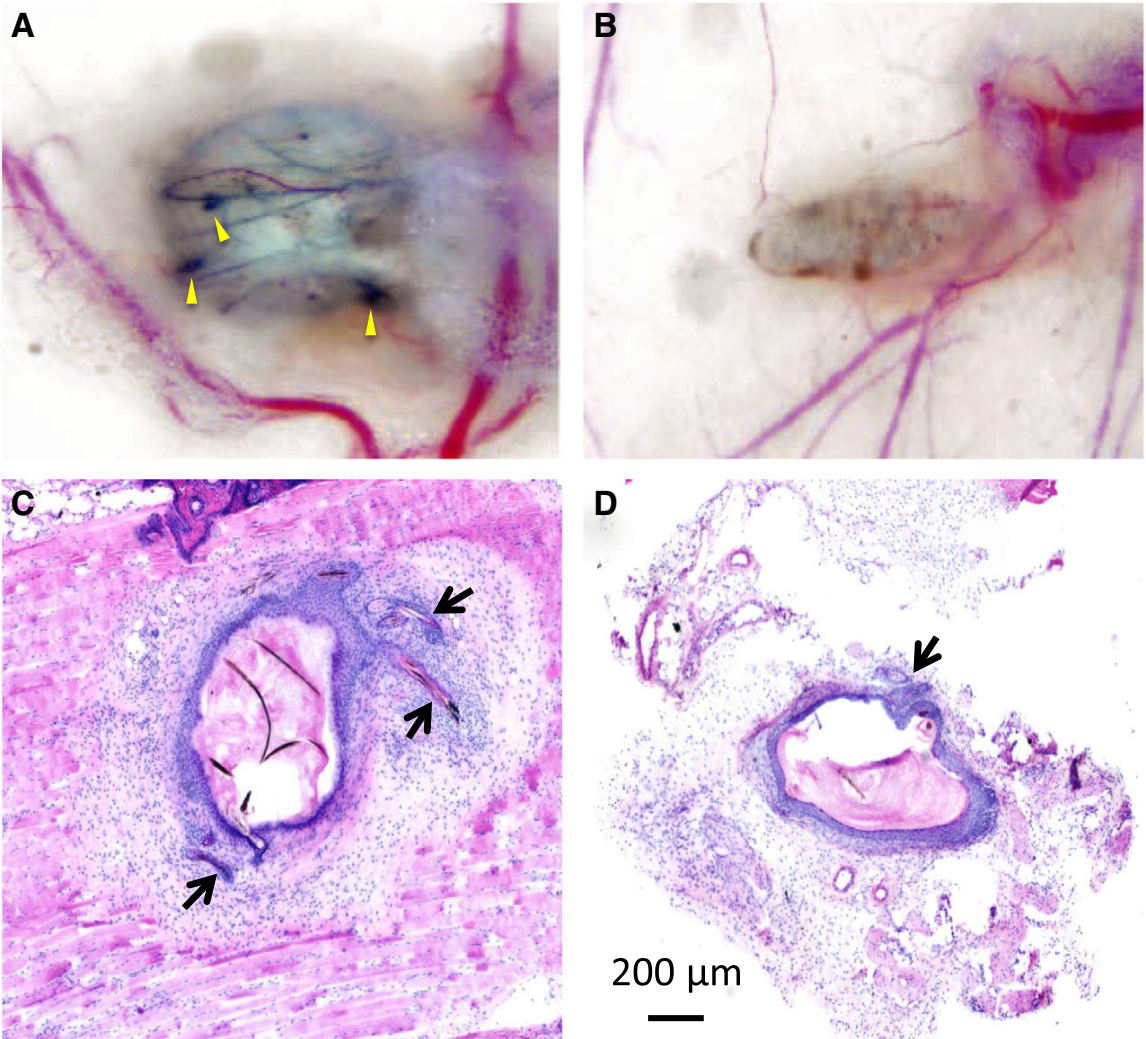
Spheroids of DPCs cultured with CAO1/2 FP recovered the hair‐forming ability. Spheroids were made from DPCs cultured with CAO1/2‐FP or DMEM and newborn‐derived keratinocytes, which were inserted into the hypodermis of host nude mice for a patch assay. Hair shafts were produced in an epithelial cyst formed with CAO1/2‐FP spheroids (A), but not with DMEM spheroids (B). Yellow arrowheads point to the hair bulbs. In the hematoxylin and eosin‐stained histological sections, typical hair follicles were detected in association with the cyst formed with CAO1/2‐FP spheroids (C). Hair follicle‐like structures were found in the cyst formed with DMEM spheroids in one out of three patches, but no hair bulb or shaft was observed (D). Arrows indicate hair follicles or follicle‐like structures. Scale bar, 200 μm.

Finally, the hair‐forming activity of the spheroids was examined using a patch assay. Mixed spheroids were prepared from keratinocytes and DPCs cultured in CAO1/2‐FP or DMEM and transplanted in the hypodermis of nude mice. Epithelial cysts were observed after 31 or 34 days of the spheroid transfer (Fig. 10A,B). Hair shafts were observed in two out of three patches induced with the CAO1/2‐FP spheroids, but no hair was found in the patches formed with the DMEM spheroids. Typical hair follicle structures with a hair bulb were formed in the epithelial cysts derived from the CAO1/2‐FP spheroids (Fig. 10C), but only hair follicle‐like structures without hair shafts were detected in histological sections of the patches formed from the DMEM spheroids (Fig. 10D). Although the produced hair shafts were small in number, there was a clear contrast between the CAO1/2‐FP spheroids and the DMEM spheroids on the hair‐forming ability (Table 1). These results suggest that DPCs are able to recover their hair‐forming activity by culturing in CAO1/2 medium supplemented with FGF2 and PDGF‐AA.

**Table 1:** Number of hairs produced in the cysts in the hypodermis of host nude mice that had been transplanted with the cell mixture of keratinocytes and DPC spheroids cultured with CAO1/2‐FP or DMEM.

Culture medium	Specimen number	Number of hair shafts
CAO1/2‐FP	1	18
	2	0
	3	8
DMEM	1	0
	2	0
	3	0

## Discussion

One of the ultimate challenges of DPC culture is to generate hair follicles *de novo* by transplanting them in the sites of hair loss; however, the hair‐inductive capacity of DPCs is perturbed during 2D monolayer culturing. Preparation of spheroids of DPCs is a solution for hair regeneration because the cells retain their original hair‐forming properties in the 3D structure formed, with appropriate ECM molecules including *vcan*. In fact, the dispersal of aggregation in DPs leads to interference in the hair follicle formation in the developmental process 20. Moreover, loss of cell–cell adhesion *in vivo* appears to initiate the DP miniaturization and androgenic alopecia 21. However, it is still difficult to maintain the cellular viability and also the hair‐forming capacity of DPC spheroids *in vitro* because of the rapid shrinkage of the spheroids during the 3D culturing. The shrinkage would cause cell death of the DPCs in the central oxygen‐poor area and disturbance in the cell–cell or cell–ECM interactions.

To overcome these problems, we had hypothesized that DPC spheroids can self‐organize their structure and functions even *in vitro* by producing appropriate species and amounts of ECM proteins, if the DPC‐specific differentiation is promoted. We have identified a better condition to maintain the properties of the DPC spheroids, in which the cells were stimulated with both the adipogenic and osteogenic factors, at a concentration lower than those used for inducing adipocytes or osteocytes from MSCs. This hypothesis was based on the observation that DPCs express the marker genes of both adipocytes and osteocytes. The CAO is a mixture of the adipogenic medium and the osteogenic medium, and the CAO1/2 contains the factors diluted to half of that in CAO. Because CAO1/2 was better than CAO for DPC‐related gene expression (Fig. 2), it appears that the differentiation toward DPC lineage requires a relatively weaker level of stimulation than the optimal levels for the adipogenic or osteogenic lineage. In fact, the cells cultured in CAO1/2 have neither Oil Red O‐positive lipid droplets nor Alizarin Red S‐positive calcium deposition, which are typical characteristics for adipocytes and osteocytes, respectively. CAO1/2 contains both the osteogenic and adipogenic factor dexamethasone; two osteogenic inducers, β‐glycerol phosphate and ascorbic acid; and two adipogenic additives, IBMX and insulin. In addition, CAO1/2 supplemented with FGF2 and PDGF‐AA avoided the spheroid shrinkage (Figs 3 and 4) and enhanced the expression of the DPC‐related genes (Figs 5 and 6) and proteins (Figs 7, 8, 9). Thus, the combination of these factors at appropriate doses would be critical to exert and maintain the DPC‐specific properties.

Although the exact roles of the factors used in this study are not yet uncovered, dexamethasone induces Runx2 expression by FHL2/β‐catenin‐mediated transcriptional activation. The enhanced Runx2 activity leads the adipose‐derived stem cells to secrete collagen type I 22. β‐Glycerol phosphate provides phosphate to produce hydroxyapatite in the osteocyte precursor cells and also influences intracellular signaling molecules 22. IBMX is a nonselective phosphodiesterase inhibitor and raises intracellular cAMP and protein kinase A, which activates the transcription factor peroxisome proliferator‐activated receptor γ (PPARγ) required for adipogenic gene expression 23. Insulin stimulates deoxyglucose uptake and glycolysis. Because the targeted deletion of PPARγ in follicular stem cells in mice causes a scarring alopecia‐like phenotype 23, IBMX‐mediated PPARγ expression may support the gene expression required for hair formation. Ascorbic acid appears to enhance the stemness of adipose‐derived stem cells by stimulating collagen synthesis through the extracellular signal‐regulated kinase 1/2 pathway. The adipose‐derived stem cells treated with ascorbic acid promote differentiation toward endothelial and epidermal lineages in the cutaneous wound tissue 24.

DPCs change their gene expression profile during the hair cycle, which is closely related to the transition of DP between the active and quiescent modes. For example, *alpl* expression is stronger in the early anagen DP, gradually decreases from the bottom of the DP in the mid‐anagen phase, and rapidly diminishes in the catagen phase 18. Some DPCs and dermal sheath cells also express *sox‐2*, which is expressed in various precursor cells, such as the MSCs. These facts suggest that the DP is composed of heterogeneous cells, and the property of each DPC may greatly change during the hair cycle depending on the microenvironment, which partially explains why we could restore the DPC‐specific properties in the 3D culture by stimulation with FGF2 and PDGF‐AA, in addition to the adipogenic and osteogenic factors. FGF2 increased the volume of the spheroids in a dose‐dependent manner, but PDGF‐AA was not effective on the volume enlargement, at least not in a range from 20 to 100 ng·mL^−1^ (Fig. 4A). Because the inoculated cell number was the same as that in the spheroids, the difference in the spheroid volume would mainly be due to the difference in the amount of the ECM molecules in the spheroids. In addition, GFP fluorescence, which is a good indication of *vcan* expression level, also increased upon stimulation with FGF2 in a dose‐dependent manner (Fig. 4B). Interestingly, PDGF‐AA could synergistically act with FGF2 and increase the spheroid volume to more than that when stimulated with FGF2 only, although PDGF‐AA alone did not show any effect on spheroid enlargement as mentioned above. Thus, one of the roles of FGF2 should be to stimulate ECM molecule production including *vcan*, which is important for providing hair‐inductive property to the DPCs. It is known in a study of idiopathic pulmonary fibrosis that both FGF and PDGF‐AA receptors cooperatively promote differentiation of fibroblasts into myofibroblasts, which leads to excessive deposition of ECM proteins in the interstitial space 25. Dermal sheath cells are closely related to myofibroblasts because both cell types vigorously express the *asma* gene. They are thought to be reservoirs of DPCs that may be partially replaced in early anagen phase to recover their hair‐inducing activity and to change hair thickness 26. Thus, FGF and PDGF‐AA may also support DPC properties through the expression of the myofibroblast‐related genes including *asma*.

DPCs share the expression of characteristic genes in other cell types, such as leptin in adipocytes and *opn* in osteocytes. It is not surprising because DPCs, as well as adipocytes and osteocytes, are thought to be derived from similar precursors. Iguchi *et al*. 27 reported that human DPCs produced leptin, but a monolayer culture of DPCs in adipogenic medium did not induce any lipid vacuoles, suggesting that unknown autocrine factors of DPCs may inhibit their differentiation toward the adipocyte lineage 27. *opn* is expressed in MSCs cultured in the osteogenic medium, which has a versatile role in the cell–ECM interaction and anti‐inflammation in osteoblasts and osteoclasts. Although the function of *opn* is less clear in DPCs, its expression is found at a certain level in DPs during the entire hair cycle and increases in shrunk DPs of catagen hair follicles, which may promote the formation of a tightly aggregated DP and protect the DPCs from apoptosis that might be induced by cytokines or hypoxia during the catagen phase 28.

It has been previously reported that FGF2 has mitotic activity on dermal fibroblasts *in vitro* 29 and stimulates the cell proliferation of DPCs derived from sheep hair follicles 30, although proliferation seldom occurs in DPCs *in vivo*. PDGF‐AA is one of the key molecules secreted from adipose precursor cells in the dermis as a paracrine factor, which promotes hair follicle development and hair growth 31. We cultured DPCs with FGF2 and PDGF‐AA in CAO1/2 medium as a monolayer culture for only 3–4 days before preparing the spheroids, because long‐term cultivation might induce apoptosis and reduce ALPL activity 32. CAO1/2‐FP spheroids could be maintained without shrinkage for 4 days (Figs 3 and 4A) and express *vcan*, *asma*, *alpl* and *opn* genes to levels nearly comparable with those in the intact DPCs (Figs 5 and 6). Furthermore, the CAO1/2‐FP spheroids synthesized ASMA and ALPL proteins at a higher level (Figs 7, 8, 9) and could recover their hair‐forming activity (Fig. 10). The DPCs in the DMEM spheroids showed a tendency to dissociate during the immunocytochemical treatment, which suggests that CAO1/2‐FP could strengthen the cellular cohesion in the spheroids. Although a part of these effects should be explained by enrichment of ECM proteins, other molecules induced by the factors would be required to exert the full function of the active DP. In this study, we have found the way to promote and sustain the DPC‐specific property *in vitro*, which allows us to compare comprehensively the DPCs that lost the hair‐forming activity and those that recovered the activity in the future.

## Conclusions

CAO1/2 medium consisting of adipogenic and osteogenic inducers can promote the expression of *vcan* in DPC spheroids, which is closely related to the hair‐inductive property, and this medium when supplemented with FGF2 and PDGF‐AA was able to prevent spheroid shrinkage, in addition to stimulation of the expression of DPC‐related genes and recovery of the hair‐forming activity. These facts indicate that adipogenic and osteogenic factors, combined with FGF and PDGF‐AA, would induce the self‐organization of DPCs in 3D culture, to make the structure and functions similar to that in intact DPs, by sustaining appropriate cell–cell and cell–ECM interactions to exert the hair‐forming activity.

## Conflict of interest

The authors declare no conflict of interest.

## Author contributions

TK mainly carried out laboratory work, data analysis and drafting the manuscript. IN performed part of the experiments and data analysis. IN and AN took part in the manuscript writing. TM conceived and supervised the study and reviewed the manuscript.
